# External Quality Assessment of Reading and Interpretation of Malaria Rapid Diagnostic Tests among 1849 End-Users in the Democratic Republic of the Congo through Short Message Service (SMS)

**DOI:** 10.1371/journal.pone.0071442

**Published:** 2013-08-13

**Authors:** Pierre Mukadi, Philippe Gillet, Albert Lukuka, Joêl Mbatshi, John Otshudiema, Jean-Jacques Muyembe, Jozefien Buyze, Jan Jacobs, Veerle Lejon

**Affiliations:** 1 Institut National de Recherche Biomédicale, Kinshasa, Democratic Republic of the Congo; 2 Université Pédagogique Nationale, Kinshasa, Democratic Republic of the Congo; 3 Department of Clinical Sciences, Institute of Tropical Medicine, Antwerp, Belgium; 4 Laboratoire National de Référence Paludisme (Programme National de Lutte contre le Paludisme), Kinshasa, Democratic Republic of the Congo; 5 Programme National de Lutte contre la Tuberculose, Kinshasa, Democratic Republic of the Congo; 6 Integrated Health Project - United States President’s Malaria Initiative, Kinshasa, Democratic Republic of the Congo; 7 Université de Kinshasa, Kinshasa, Democratic Republic of the Congo; 8 Institut de Recherche pour le Développement, UMR 177 IRD-CIRAD INTERTRYP, Campus International de Baillarguet, Montpellier, France; Kenya Medical Research Institute - Wellcome Trust Research Programme, Kenya

## Abstract

**Background:**

Although malaria rapid diagnostic tests (RDT) are simple to perform, they remain subject to errors, mainly related to the post-analytical phase. We organized the first large scale SMS based external quality assessment (EQA) on correct reading and interpretation of photographs of a three-band malaria RDT among laboratory health workers in the Democratic Republic of the Congo (DR Congo).

**Methods and Findings:**

High resolution EQA photographs of 10 RDT results together with a questionnaire were distributed to health facilities in 9 out of 11 provinces in DR Congo. Each laboratory health worker answered the EQA by Short Message Service (SMS). Filled-in questionnaires from each health facility were sent back to Kinshasa. A total of 1849 laboratory health workers in 1014 health facilities participated. Most frequent errors in RDT reading were i) failure to recognize invalid (13.2–32.5% ) or negative test results (9.8–12.8%), (ii) overlooking faint test lines (4.1–31.2%) and (iii) incorrect identification of the malaria species (12.1–17.4%). No uniform strategy for diagnosis of malaria at the health facility was present. Stock outs of RDTs occurred frequently. Half of the health facilities had not received an RDT training. Only two thirds used the RDT recommended by the National Malaria Control Program. Performance of RDT reading was positively associated with training and the technical level of health facility. Facilities with RDT positivity rates >50% and located in Eastern DR Congo performed worse.

**Conclusions:**

Our study confirmed that errors in reading and interpretation of malaria RDTs are widespread and highlighted the problem of stock outs of RDTs. Adequate training of end-users in the application of malaria RDTs associated with regular EQAs is recommended.

## Introduction

Although malaria RDTs are relatively simple to perform, they remain subject to errors [1]. Many errors are related to the post-analytical phase, *i.e.* wrong reading of the test and control lines and incorrect interpretation of the results [1], [2]. External quality assessment (EQA) provides insights into performance levels of end-users and allows the assessment of the type and frequency of errors. However, delivery of high quality EQA panels to numerous RDT end-users may prove logistically challenging, especially in resource poor regions such as in many sub-Saharan countries, where malaria is endemic. Photographed RDT results may provide an alternative. Photographs have been used to assess the interpretation proficiency of HIV RDT performers [3] and to assess whether trained end-users of malaria RDTs had maintained their performance of accurate RDT reading [4]. However, large scale photograph-based EQAs assessing performance of end-users in reading and interpretation of malaria RDTs have not yet been organised.

In the last decade, Africa has witnessed an expansion in mobile phone use. Short message service (SMS) offers a cheap, simple and attractive mean of communication, access to large numbers of mobile telephone users and rapid data flow, also in a medical context. Examples of SMS based health interventions in Africa include projects on HIV/AIDS prevention [5], improved adherence to anti-retroviral therapy [6], [7], accelerated communication of medical results [8] and accelerated communication of the malaria incidence and of stock data of malaria drugs [9], [10]. Substitution of paper based reporting by SMS is expected to reduce data entry mistakes and may improve malaria control [11].

We here describe the results of an EQA on reading and interpretation of a three-band malaria RDT among laboratory health workers in the Democratic Republic of the Congo (DR Congo), a malaria endemic country where malaria RDTs have been introduced since 2010. The EQA consisted of photographed results of the RDT recommended by the National Malaria Control Program (Programme National de Lutte contre le Paludisme, PNLP). Individual laboratory health workers were invited to reply by SMS and a questionnaire was completed by the laboratory supervisor of the targeted health facilities.

## Materials and Methods

### Ethics Statement

The EQA followed an interpretive proficiency test scheme, was set up within the framework of routine diagnostics and capacity building purposes and was coordinated by the Institut National de Recherche Biomédicale (INRB), which is the reference laboratory of the PNLP. As indicated in ISO/IEC 17043∶2010(E) (Conformity assessment – General requirements for proficiency testing, edition 2010, International Organization for Standardization, ISO Committee on Conformity Assessment, Geneva, Switzerland), the identity of participants was known only to people involved in coordinating the EQA programme, and was maintained confidential. Participation of laboratory health workers to the EQA was on a voluntary basis and their privacy was strictly respected. Following ISO/IEC 17043∶2010(E), the Declaration of Helsinki, the Council for International Organizations of Medical Sciences (CIOMS) guidelines for epidemiological research, the guidelines of the American Anthropological Association, as well as the Belgian law of May 7, 2004 concerning experiments on the human person, and taking into account that all results of EQAs in Belgium are publicly available via the internet, submission of this EQA to the Institutional Review Board of the Institute of Tropical Medicine was deemed unnecessary.

### Design of the Study

The EQA took place from May till July 2012 in DR Congo. It consisted of (i) 1 set of photographs with multiple choice questions (MCQ) for each laboratory health worker; (ii) 1 questionnaire per health facility and; (iii) leaflets explaining the EQA in detail and giving instructions on how to answer to the MCQ by SMS. The study coordinator at INRB shipped in total 2305 photographs and 1176 questionnaires to 14 co-investigators in 9/11 provinces in DR Congo. Provincial co-investigators were selected based on convenience, accessibility and personal contacts with the study coordinator or the PNLP. Co-investigators belonged to the National Tuberculosis Control Program, the Provincial Division of Ministry of Health and the Integrated Health Project (PROSANI). They distributed the photographs to laboratory health workers using malaria RDTs, and the questionnaires to the laboratory responsible of each corresponding health facility and gave the laboratory health workers a basic explanation about the study. Participating health facilities were members of the network of the National Tuberculosis Control Program, of PROSANI or of INRB, were provided with malaria RDTs by the PNLP or other organisations and were chosen by the co-investigators based on convenience, accessibility and personal contacts. Laboratory health workers answered the MCQ individually, by sending an SMS to the coordinator. Filled-in questionnaires were collected from the laboratory responsible by the co-investigators and shipped back to the study coordinator.

### EQA Photographs with Multiple Choice Questions

**Figure 1:**
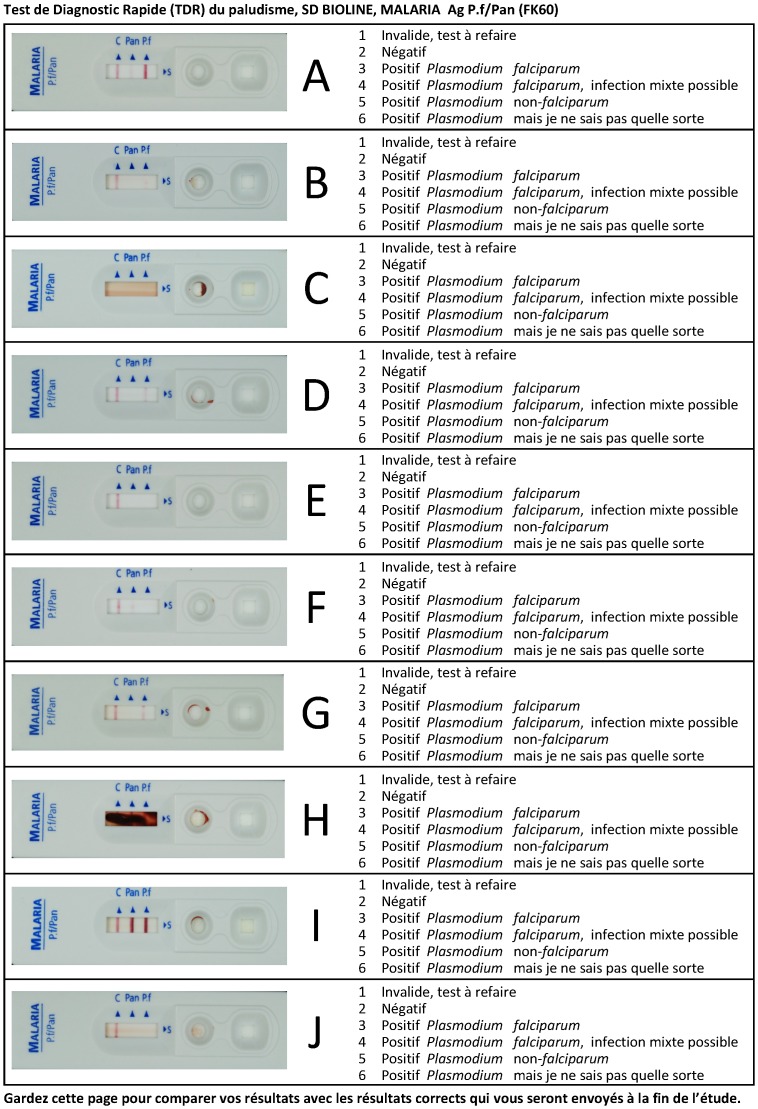
Photographs of 10 SD malaria Ag Pf/Pan (Standard Diagnostics Inc) rapid diagnostic test results with multiple choice questions.

Ten SD malaria Ag Pf/Pan (HRP2/pLDH, Standard Diagnostics, Inc., Kyonggi-do, Korea) test results were photographed (Figure 1). Photographs were printed in real size on photographic paper (maco silk normal full colour CMYK, 300dpi, Bulckens, Herenthout, Belgium) and next to each photograph, a MCQ was provided. For each MCQ, six options were listed, numbered from 1 to 6. Per MCQ, only one option was considered correct. The SMS reply contained the following information (i) the code “Eeq” (for “Evaluation Externe de la Qualité”), (ii) a 10 digit answer code consisting of the combination of the options (number from 1 to 6) for each of the 10 MCQs, (iii) the participant’s name and first name, (iv) the health facility of the participant and (v) province. Participants were transferred 1 U.S. $ of phone credit. After closure of the EQA, an SMS message with the correct options for all MCQs was sent to all EQA participants.

### Questionnaires

The questionnaire was filled in by the laboratory responsible. It consisted of closed questions about health facility type (categorized as provincial laboratory, hospital, health centre and health post), conditions of RDT and microscopy use, brand of RDT used, time of experience with RDTs, duration of stock ruptures in 2011, any trainings on the use of malaria RDTs received, and elapsed time since this training. For some questions, the option “other” could be further specified by open answers. Open questions assessed the number of consultations, the numbers of RDTs performed in April 2012 (the month preceding the EQA) and their positivity rate. For each health facility, a list of laboratory health workers was requested, which served as a unique identifier to match the results of the facility questionnaire with those of the MCQ.

### Data Entry and Analysis

SMS replies were transferred by Bluetooth to an Excel database managed by the study coordinator. If more than one SMS per laboratory health worker was received, only the first SMS was considered, which means that there was no system that could allow the laboratory health worker to re-call or correct a SMS sent with errors that he/she got aware of later. Ineligible answer codes shorter or longer than 10 digits or containing numbers higher than 6 were removed. To express competence, answers were scored as 1, 0.5 and 0 when they were categorized as correct or as minor and major error respectively (Table 1), and the sum of all scores was made (maximal score of 10). The Mc Nemar chi square test was used to compare proportions of correct MCQ answers between different questions.

**Table 1:** Composition of EQA and percentage of answers received (N = 1849) for each photograph.

		Line			% of answers (N = 1849)					
Photo number	Correct answer	Control	Pan	*P.f.*	1. Invalid	2. Negative	3. Positive, *P. falciparum*	4. Positive, *P. falciparum,* mixed infection possible	5. Positive, *P.* non*-falciparum*	6. Positive, but species not known
A	Positive, *P. falciparum,* mixed infection possible	+	W	S	1.8**	0.8**	32.8*	58.1	1.9**	4.6*
B	Positive, *P. falciparum*	+	−	F	3.2**	31.2**	58.4	1.9*	2.3**	3.0*
C	Invalid	−	−	−	86.7	9.0**	1.6**	1.2**	0.5**	0.9**
D	Positive, *P. falciparum*	+	−	W	2.9**	4.1**	81.2	4.0*	4.5**	3.2*
E	Negative	+	−	−	2.8**	90.2	2.2**	1.6**	1.7**	1.5**
F	Positive, *P.* non*-falciparum*	+	W	−	5.2**	13.1**	6.5*	4.9*	53.7	16.5*
G	Positive, *P. falciparum*	+	−	M	2.9**	2.1**	82.9	5.6*	3.0**	3.6*
H	Invalid	BG	BG	BG	67.5	4.4**	2.3**	6.7**	7.7**	11.4**
I	Positive, *P. falciparum,* mixed infection possible	+	S	S	2.6**	2.3**	10.3*	77.7	1.9**	5.2*
J	Negative	+	−	−	5.4**	87.2	2.8**	1.4**	1.4**	1.8**

− : negative,+positive, F: Faint positive, W: Weak positive, M: medium positive, S: Strong positive, BG: strong background, lines invisible.

* minor error,

** Major error.

Results of laboratory questionnaires which could be matched to at least one SMS, were entered in an epi-info database (Epi-info™, CDC, Atlanta, USA). The excel and epi-info databases were fused to make the link between the EQA score and health facility characteristics. To avoid correlation within health facility, one representative laboratory health worker for each health facility was selected (first one alphabetically). For bivariate analysis of MCQ scores associated with health facility properties, comparisons of median scores between 2 groups were performed using the Mann-Whitney Rank Sum Test. For multiple analysis of MCQ scores associated with health facility properties, a backward median regression model with MCQ score as outcome and the health facility properties from bivariate analysis as independents was built. For the covariates with missing values a missing indicator was added.

## Results

### EQA

**Figure 2:**
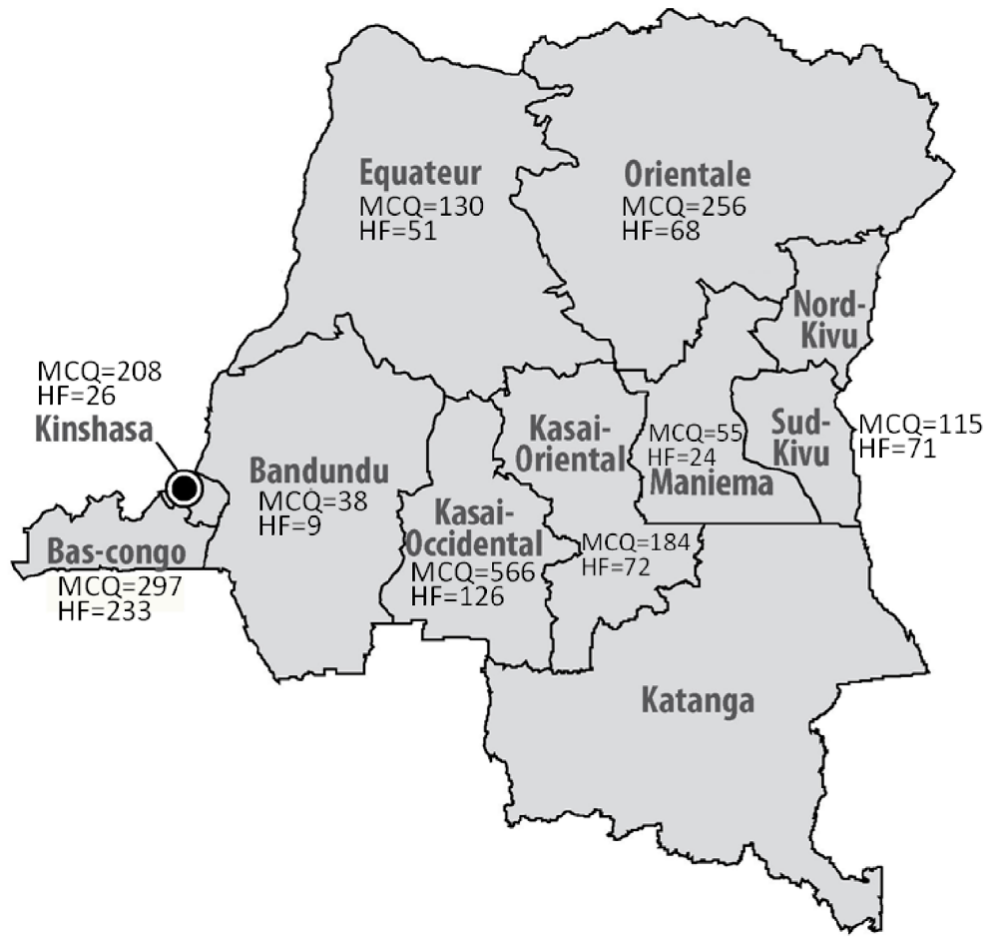
Location of 1849 eligible MCQ answers (MCQ) and of 680 health facilities (HF) participating in the EQA.

The study coordinator received 2484 SMS replies from 1892 laboratory health workers. After removal of 592 duplicates, 1849 answers (97.7%, 1849/1892) from 1014 health facilities were eligible, corresponding to 80.2% (1849/2305) of shipped photographs. The EQA was closed after 66 days and median time of SMS receipt was 21 days (interquartile range IQR 12–35 days). Most SMS (30.6%) originated from the province of Kasai Occidental (Figure 2).

For each photograph, between 53.7 and 90.2% of correct answers were received (Table 1). Some errors occurred frequently. Invalid RDT results (photographs C and H) were not recognized by respectively 13.2 and 32.5% of participants, with 7.4% of participants missing the invalid result in both MCQs. Negative RDT results (photographs E and J) were not recognized by respectively 9.8 and 12.8% of participants, 5.6% of participants failed twice to recognize a negative result. Single faint or weak test lines (Photographs B, D and F) were reported as negative by respectively 31.2, 4.1 and 13.1% of participants and all three photographs were reported negative by 1.4% of participants. Test lines of weak intensity next to the control line appeared to be more frequently overlooked (9.0% more negative readings of photograph F than of D). Comparison of photographs with identical patterns but different line intensities further evidenced overlooking of low intensity lines: there were more correct answers for photographs I compared to A (*p*<0.0001) and for photograph G compared to B (*p*<0.0001). Participants encountered difficulties in discriminating *Plasmodium falciparum* from non-*falciparum*, and recognizing mixed infections. Photographs G and I, were read as positive but had wrong or no species identification for 12.1 and 17.4% of participants respectively (5.2% had wrong or no species identification for both photographs).

**Figure 3:**
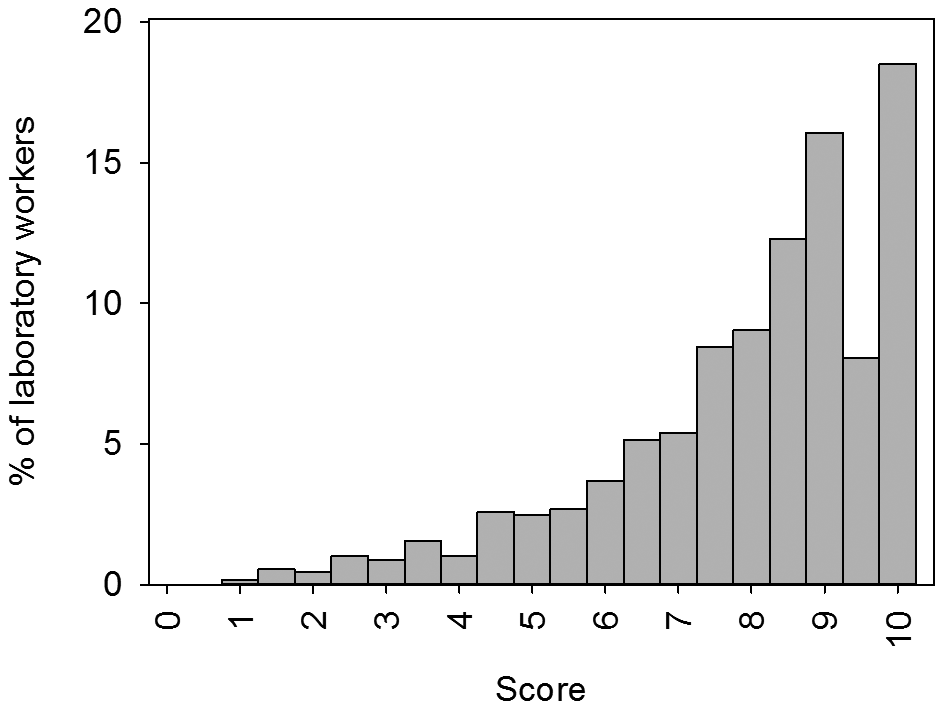
Competence of individual end-users to read and interpret RDT test results expressed as a score on 10.

The median competence score for the 1849 laboratory health workers was 8.5/10 (IQR 7–9.5, Figure 3). All results were correctly reported (score 10/10) by 18.5% of end-users and satisfying scores of 9.5 and 9 were obtained by another 24.1% of end-users. 29.9% did not make any “major” error. Although no one scored zero, poor scores lower than 6.5 were obtained by 22.2% of participants.

### Questionnaire

In total, 859 completed questionnaires were received, out of which 179 could not be matched to a SMS, thus 680 questionnaires (79.2%) were considered, corresponding to 1299 SMS replies (68.5%) and to 57.8% of shipped questionnaires (680/1176). Most questionnaires came from Bas-Congo (333/680, 34.3%, figure 2). Among the health facilities, health centers were most represented (63.1%, Table 2).

**Table 2:** Characteristics of 680 health facilities, and circumstances of microscopy and malaria RDT use.

	Health post	Health centre	Reference health centre	Hospital	Laboratory and supervising	Total
Number	80	429	90	70	11	680
Microscopy						
No reply	2.5%	3.3%	1.1%	0.0%	0.0%	2.5%
No	56.3%	45.0%	10.0%	2.9%	9.1%	36.8%
Yes	41.3%	51.7%	88.9%	97.1%	90.9%	60.7%
Always	13.8%	22.6%	58.9%	61.4%	72.7%	31.2%
RDT stock out	13.8%	11.0%	3.3%	2.9%	9.1%	9.4%
Malaria suspicion with negative RDT	7.5%	8.6%	7.8%	2.9%	0.0%	7.6%
Request from clinician	3.8%	4.0%	12.2%	24.3%	0.0%	7.1%
Other or not specified^a^	2.5%	5.6%	6.7%	5.7%	9.1%	5.4%
RDT use						
No reply	0.0%	2.1%	0.0%	0.0%	0.0%	1.3%
No	2.5%	14.0%	18.9%	15.7%	36.4%	13.8%
Yes	97.5%	83.9%	81.1%	84.3%	63.6%	84.9%
Always	78.8%	52.9%	42.2%	27.1%	27.3%	51.5%
Request from clinician	5.0%	4.2%	11.1%	35.7%	9.1%	8.5%
Giemsa stock out or no microscope	3.8%	6.5%	7.8%	4.3%	9.1%	6.2%
If RDT available^b^	5.0%	6.5%	5.6%	4.3%	9.1%	6.0%
RDT in ward, microscopy in lab	2.5%	4.7%	4.4%	5.7%	0.0%	4.4%
Malaria suspicion with negative TBF	0.0%	3.3%	5.6%	4.3%	0.0%	3.2%
Other or not specified	2.5%	5.8%	4.4%	2.9%	9.1%	5.0%
Training						
No reply	0.0%	4.0%	3.3%	2.9%	0.0%	3.2%
No	70.0%	39.2%	50.0%	38.6%	72.7%	44.7%
Yes	30.0%	56.9%	46.7%	58.6%	27.3%	52.1%
Consultations/month, median (IQR)^c^	25 (18–33)	130 (56–308)	201 (86–338)	304 (157–711)	40 (26–50)	30 (8–80)
RDT/month, median (IQR)^c^	11 (5–22)	40 (13–90)	40 (13–106)	50 (6–114)	97 (30–112)	35 (11–88)

TBF Thick blood film; RDT rapid diagnostic test.

a besides facilities not specifying the circumstances of use, includes the options: (i) RDT in ward, microscopy in laboratory; (ii) patient follow-up; (iii) person performing the RDT absent; (iv) other.

b under the option “other”, 41/47 health facilities indicated themselves to perform RDTs if they were available.

c in April 2012.

Almost all hospitals (n = 70) performed microscopy and 61.4% used microscopy for all suspected cases. This contrasts with the health posts (n = 80), where 41.4% performed microscopy, and only 13.8% always did. However, almost all (97.5%) health posts declared to perform RDTs, and 78.8% do so always. Among hospitals, 84.3% used RDTs, but only 27.1% always. Specific circumstances and applications of RDTs included request by the clinician (8.5%, 58/680), use of RDT in the ward versus and microscopy in the lab (4.4%, 30/680), unavailability of Giemsa stain or a functional microscope 6.2% (42/680) and suspicion of malaria but negative microscopy 3.2% (22/680). Under “other” circumstances for RDT use 41/680 health facilities (6.0%) specified spontaneously to use RDTs if they were available. Indeed, in 2011 stock outs had occurred in 62.2% (226/598) of health facilities, and lasted in half of cases from 1 to 3 months or longer.

Four different malaria RDT brands were used (not specified for 11.5% of health facilities): (i) Paracheck Pf-Rapid Test (Orchid Biomedical Systems, Goa, India, 77/680, 11.3%); (ii) SD malaria Ag Pf/Pan (394/680, 57.9%) which is the RDT actually recommended by the PNLP; (iii) SD Malaria antigen Pf/Pv (Standard Diagnostics, Inc., Kyonggi-do, Korea, 99/680, 14.6%) and; (iv) SD Malaria antigen Pf (32/680, 4.7%). SD Malaria antigen Pf/Pv exclusively circulated in Kasai Occidental and Sud Kivu where it was used in half of the participating health facilities (respectively 62/126 and 37/71), while in Maniema only Paracheck Pf-Rapid Test was used (22/24).

RDTs were being used since a median time of 12 to 24 months. Training on the use of malaria RDTs was done for 52.1% of facilities (354/680, Table 2), and median time since training ranged between 12 and 24 months. However, 70.0% of health posts were not trained (56/80). In April 2012, health facilities had used a median of 35 RDTs (IQR 11–88) with a median positivity rate of 48.5% (IQR 28–68%).

### MCQ Scores in Function of the Results of the Questionnaire

In bivariate analyses, the region of the health facility, the health facility level, the RDT brand and the RDT positivity rate were significantly related to the MCQ score (Table 3). Training, use of RDTs, time of experience with RDTs, and number of RDTs run per month did not influence the MCQ score.

**Table 3:** Bivariate analysis of MCQ scores associated with health facility properties.

Health facility property, median score and IQR		*p*
Region	Eastern Congo (n = 163: Oriental, Sud-Kivu, Maniema): median 8, IQR 6.5–8	0.005
	Rest of Congo (n = 517): median 8.5, IQR 7–9.5	
Health facility level	High (n = 81: hospital, laboratory, supervising): median 9.0, IQR 8.0–10	0.003
	Low (n = 609: health post, health center, referral health center): median 8.5, IQR 7–9.5	
RDT brand	SD malaria Ag Pf/Pan (n = 394): median 8.5, IQR 7.5–9.5	<0.0001
	Other (n = 208): median 8.0, IQR 6.5–95	
RDT positivity rate	≤50% (n = 225): median 8.5, IQR 7.5–9.5	0.02
	>50% (n = 213): median 8.5, IQR 7–9	
Training	Yes (n = 354): median 8.5, IQR 7.5–9	0.3
	No (n = 304): median 8.5, IQR 6.5–9.5	
Use of RDT	Yes (n = 577): median 8.5, IQR 7–9	0.4
	No (n = 94): median 8.5, IQR 7–9.5	
Experience with RDT	>1 year (n = 364): median 8.5, IQR 7.0–9.5	0.25
	<1 year,(n = 269): median score 8.5 IQR 7–9.5	
Number of RDT/month	>35 (n = 258): median 8.5, IQR 7–9	0.7
	≤35 (n = 287): median 8.5, IQR 7–9.5	

In multiple quantile regression, the final model was Median(Score|T,Tm,L,P,Pm,E) = 8.5+0.5*T+1*Tm+1*L-0.5*P-0.5*Pm-1*E (With T = 1 for training and 0 otherwise, Tm = 1 for information on training missing and 0 otherwise, L = 1 for high level and L = 0 for low level, P = 1 for RDT positivity rate >50%, P = 0 otherwise and Pm = 1 for information on RDT positivity rate missing, 0 otherwise, and E = 1 for Eastern Congo, 0 otherwise). No interactions were significant.

For health facilities of the same level, from the same region and with the same positivity rate, facilities with training had a median MCQ score 0.5 higher than those without training (*p* = 0.0002). For health facilities with same training, positivity rate and region, the median MCQ score for high level health facilities was 1 higher than for low level facilities (*p*<0.0001). For health facilities with the same training, level and region, those with a RDT positivity rate >50% had a median MCQ score 0.5 lower than for those with a positivity rate< = 50% (*p*<0.0001). Health facilities with same training, level and positivity rate located in Eastern Congo had a median MCQ score 1 lower than those not in Eastern Congo (*p*<0.0001).

## Discussion

The results of an SMS and photograph based EQA in DR Congo showed that reading and interpretation of malaria RDTs was poor for one out of four laboratory health workers. The most frequent errors were failure to recognize invalid or negative test results, overlooking of faint-intensity test lines and incorrect identification of *Plasmodium* species. Stock outs of RDTs occurred frequently. Half of the health facilities had not received RDT training, while only two thirds used the RDT recommended by the PNLP. After multiple regression, performance of RDT reading was better in comparable health facilities that received training, that were of higher level, that reported RDT positivity rates ≤50% and that were not located in Eastern DR Congo.

Some strong points of the SMS based EQA should be underlined. The low proportion of invalid results illustrates the simplicity of the SMS system already reported in other studies [11]. The power of mobile technology for EQA in a low resource setting is underlined by the number and the speed of SMS replies received in comparison with “paper” questionnaires. These findings confirm the potential of SMS based reporting to reduce turnaround time and loss rate compared to paper records [8], [12]. Moreover, the SMS-based EQA appeared less susceptible to geographical limitations compared to the paper questionnaire, confirming the benefit of an SMS based system when results have to be transmitted over greater distances [12].

The EQA identified overlooking of faint or weak test lines as the most common error in reading and interpretation of RDTs, which could –in real life - lead to false negative results. The same problem has been observed by other authors [1], [4]. Test lines, even faint, should be interpreted as positive [13]. Other previously reported errors were the interpretation of invalid tests [2], [4], [14] and lacking or incorrect species identification in a three-band test [2]. About one in ten participants interpreted negative RDTs as positive, which is lower than in EQAs for malaria microscopy [15], [16]. Health facilities reporting RDT positivity rates >50%, scored lower in the MCQ, which might be an indication for over-reporting.

Stock outs of RDTs are common in DR Congo, and this problem has been highlighted elsewhere too [17]. Avoiding stock outs therefore represents an additional challenge for RDT roll-out. SMS-based stock follow-up already has been applied for malaria drugs [9], [10], and may be extended for RDTs [18].

In DR Congo, RDTs have been introduced since 2010 [15] and since April 2012, PNLP recommends their use in all levels of the health system. At the hospital or health center level, there was no apparent uniform strategy for malaria diagnosis. They mainly applied microscopy for malaria diagnosis, but the majority also performed RDTs. In line with PNLP recommendations, nearly all participating health posts - the lowest level of health care - use RDTs for malaria diagnosis while only a minority always performed microscopy. Of note, health posts relied most on RDTs, but had received the least training and scored lowest in the EQA.

Although the SD malaria Ag Pf/Pan test is actually recommended by PNLP, only two third of health facilities use this test. The Paracheck Pf two band test was previously recommended and was still used. The use of SD Malaria antigen Pf/Pv three band test by nearly 15% of health facilities was unexpected and is inappropriate, since *Plasmodium vivax* only rarely occurs in Central-Africa [19], [20]. Use of other than the photographed test was associated with lower performance scores. This was particularly clear for Sud-Kivu and Maniema in Eastern Congo, where the recommended test was used in 16.9% and 0% of participating health facilities respectively. In addition, in Maniema only 8.3% of health facilities reported to have received training.

Improved RDT performance might be achieved through a combination of training, clear and simple bench-site job aids, supervision visits and organisation of further EQAs [1], [17], [21], [22]. Of note, almost 50% of facilities stated not to have received training on the use of malaria RDTs, which implies that in these facilities, RDTs were introduced without training. Training is considered to be an essential part of rapid test implementation and WHO recommends national programs to include training in their RDT implementation plans and budgets [1], [23]. Especially in a vast and resource poor country such as DR Congo, adequate countrywide training of health facility care workers may prove difficult, but distribution of well elaborated job aids, giving special attention to frequently made errors, might partially compensate for this. Malaria RDT job-aids and training manuals which may be down-loaded and modified as necessary are available on the WHO website [23]. Following the present EQA, PNLP and its partners organised training on the use of malaria RDTs in more than 100 health zones in DR Congo, and updated their job aids. Supervision visits, including observation of health workers and addressing in particular frequent mistakes remain recommended [1], [4] to complement an SMS based EQA system, assessing reading and interpretation performance only.

A number of limitations of this EQA should be considered. This EQA assessed only the malaria RDT reading and interpretation performance. Although this represents only one step of malaria diagnosis using a RDT, it is considered as a major source of errors [1], [2]. As discussed above, the proposed EQA system is therefore just one part of the measures to be put in place to improve malaria diagnosis. Although participants were requested to answer to the MCQ individually, collaboration cannot be excluded. Statistical analysis was therefore based on 1 participant per health facility. Collaboration to solve the MCQ might however have had an educational effect for those participants with less experience. Since we had no full control over the spread of the EQA photographs, we could not determine the exact response rate and estimations are based on the total number of photographs and questionnaires shipped by the study coordinator. Some abuse of the EQA cannot be excluded, since participants sending a SMS to the coordinator received phone credit. Payment of an incentive or use of a toll-free number is crucial for participation, since lack of telephone credit is common [9], [10]. Abuse was controlled for through listing of participants per health facility, and through verification of phone numbers and re-contacting suspect numbers. The mobile phone of the co-investigator was used to send SMS answers of participants in regions without telephone coverage (135/1849 answers from 3/14 co-investigators).

Retrospectively, the MCQ option “positive for *Plasmodium* but I do not know the species” might have been mixed-up by some participants with the option “*Plasmodium* non-*falciparum*”, since differentiation between *P. ovale*, *P. malaria* and *P. vivax* is not possible when only the pan-pLDH test line is positive. Since this option represents loss of information on *P. falciparum*, we considered it as a minor error. Conversely, presentation of the EQA answers as MCQ options might have improved overall performance and might have had an educational effect.

In conclusion, organization of further EQA’s seems indicated. It has been shown that participation to EQAs has an educational effect and raises performance, which may be explained, at least partially, by the feed-back given to the participants afterwards [16], [24]. Taking into account that spread of smartphones and internet still remain limited in resource poor settings, distribution of paper printed EQA photographs will stay necessary. However, for dissemination of EQA photographs, future EQAs may take advantage of restocking of health facilities with RDTs or anti-malaria drugs, limiting shipping costs and assuring reception. Further, the accompanying questionnaire should no longer be based on paper but rather transferred to SMS feed-back, which will increase matching between MCQ and questionnaires and decrease administrative work. In addition, an automatic feedback system to acknowledge delivery of the SMS should be foreseen [9], to avoid duplicate SMSing and anticipate the risk of health worker’s “reporting fatigue” [11]. Although network fluctuations will remain a challenge for SMS-based EQAs, the rapid expansion of mobile network coverage and decreasing costs of mobile phone services offer opportunities to adapt this SMS and photograph based EQA system to other settings and apply it for other targets, such as HIV. Furthermore, recording of basic professional data through EQA allows future access to the end-users for malaria related issues.
